# An evolving view of complex II—noncanonical complexes, megacomplexes, respiration, signaling, and beyond

**DOI:** 10.1016/j.jbc.2023.104761

**Published:** 2023-04-27

**Authors:** T.M. Iverson, Prashant K. Singh, Gary Cecchini

**Affiliations:** 1 Departments of Pharmacology, Vanderbilt University, Nashville, Tennessee, USA; 2 Departments of Biochemistry, Vanderbilt University, Nashville, Tennessee, USA; 3 Center for Structural Biology, Vanderbilt University, Nashville, Tennessee, USA; 4 Vanderbilt Institute of Chemical Biology, Vanderbilt University, Nashville, Tennessee, USA; 5 Molecular Biology Division, San Francisco VA Health Care System, San Francisco, California, USA; 6 Department of Biochemistry & Biophysics, University of California, San Francisco, California, USA

**Keywords:** complex II, succinate dehydrogenase, succinate signaling, inflammation, hypoxia, cancer metabolism, Krebs cycle, bacterial chemotaxis, respirasome, reverse electron transfer, ischemia-reperfusion, pheochromocytoma, paraganglioma

## Abstract

Mitochondrial complex II is traditionally studied for its participation in two key respiratory processes: the electron transport chain and the Krebs cycle. There is now a rich body of literature explaining how complex II contributes to respiration. However, more recent research shows that not all of the pathologies associated with altered complex II activity clearly correlate with this respiratory role. Complex II activity has now been shown to be necessary for a range of biological processes peripherally related to respiration, including metabolic control, inflammation, and cell fate. Integration of findings from multiple types of studies suggests that complex II both participates in respiration and controls multiple succinate-dependent signal transduction pathways. Thus, the emerging view is that the true biological function of complex II is well beyond respiration. This review uses a semichronological approach to highlight major paradigm shifts that occurred over time. Special emphasis is given to the more recently identified functions of complex II and its subunits because these findings have infused new directions into an established field.

Respiratory complex II (Fig. 1*A*, succinate dehydrogenase (SDH), canonically SDHA-SDHB-SDHC-SDHD, but with exceptions) is a heterotetrameric membrane-spanning enzyme first described in 1909 (1) and studied in its purified form for around a century. It has been long established that complex II is a key player in multiple respiratory processes (2, 3). The first described role of complex II was as a bioenergetic complex catalyzing two distinct redox reactions in mitochondrial aerobic respiration (Fig. 1, *B* and *C*) (2, 3, 4). Here, complex II links oxidative phosphorylation (Fig. 1*D*) with the Krebs cycle (Fig. 2). Because of the energetics of aerobic respiration, complex II works in a defined catalytic direction under aerobic conditions where the enzyme oxidizes succinate to fumarate and concomitantly reduces the high potential ubiquinone to ubiquinol (Fig. 1*C*). Under anaerobic conditions with fumarate as the terminal electron acceptor, bacterial complex II homologs can proficiently perform the reverse reaction, *i.e.*, the enzyme reduces fumarate to succinate and concomitantly oxidizes lower potential quinones such as menaquinone or rhodoquinone (5, 6, 7, 8). “Reverse” complex II activity has also been demonstrated in both mammalian mitochondria (9, 10) and bacteria (11). This reverse activity occurs under conditions where the quinone pool is highly reduced and where the fumarate concentration is sufficient to affect the thermodynamic driving force of the reverse reaction (12).

**Figure 1:**
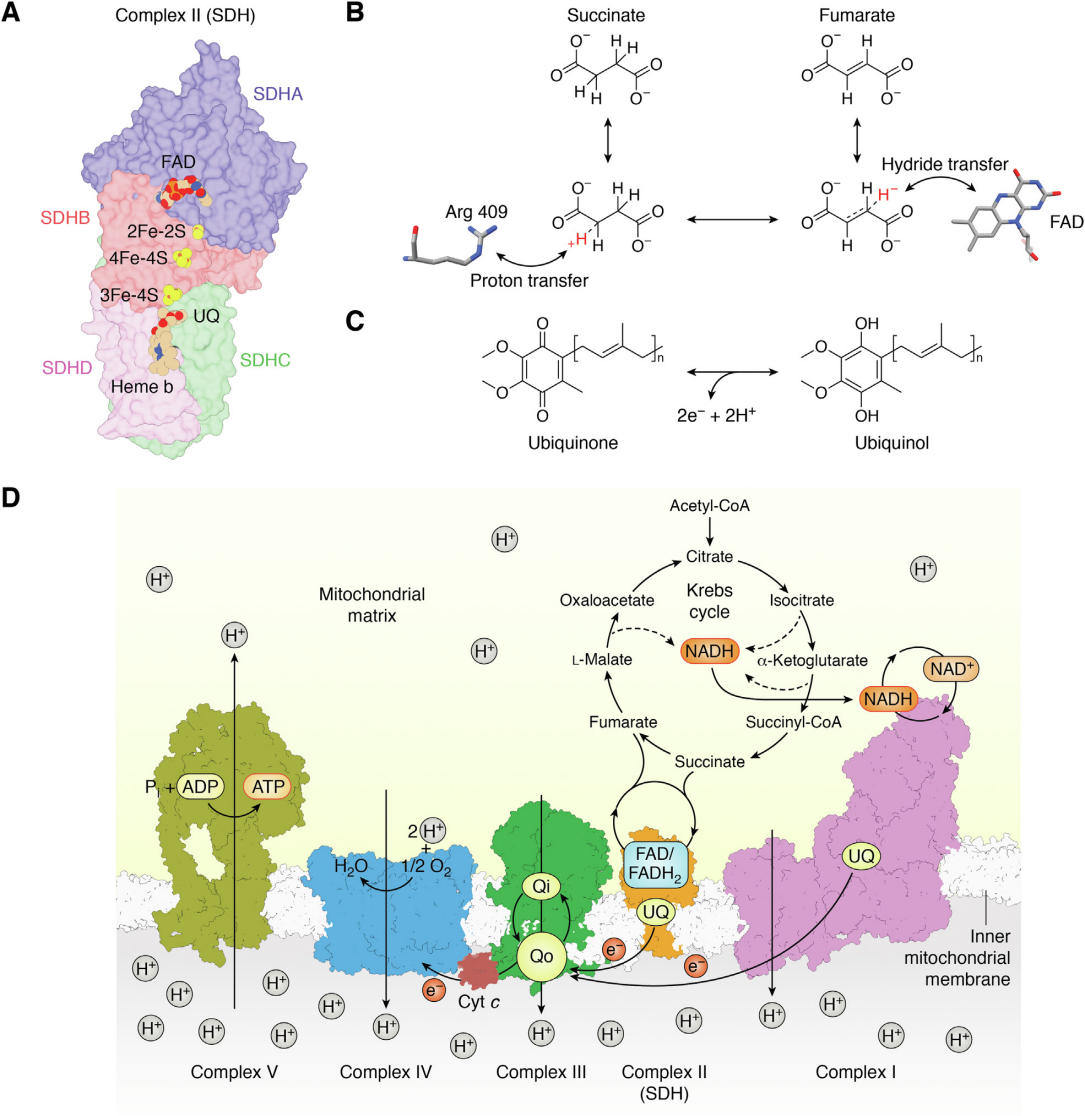
**Mitochondrial complex II and its reactions in mitochondrial aerobic respiration.***A*, architecture of mitochondrial complex II. Mitochondrial complex II (PDB 1ZOY (75)) contains four subunits: SDHA, SDHB, SDHC, and SDHD. SDHA (*blue*) is also called the flavoprotein (Fp) subunit; it houses the covalent FAD and the succinate oxidation site. SDHB (*red*) is also called the iron–sulfur (Ip) subunit; it houses the three Fe-S clusters that support electron transfer between the two active sites. The SDHC (*green*) and SDHD (*purple*) subunits are membrane-embedded, contain *b-*type heme, and house the ubiquinone reduction site. *B* and *C*, complex II couples two redox reactions: succinate/fumarate interconversion and quinone/quinol interconversion. The reactions are believed to be dependent as electrons that are coproducts of one reaction are transferred to the second active site to be used as cosubstrates. *B*, Succinate/fumarate interconversion. The mechanism of succinate/fumarate interconversion is better studied in the fumarate reduction direction. For fumarate reduction, substrate binding involves two active site histidines (His 232 and His 355, from the *E. coli* QFR) and one active site arginine (Arg 390 of *E. coli* QFR, which is equivalent to Arg 409 of human SDHA) (58). Catalysis requires an initial transfer of a hydride from FAD to fumarate *via* a neutral flavin semiquinone (71). This is followed by the transfer of a proton by an active site arginine (Arg 287, *E. coli* QFR numbering) (72, 207). Supporting the reaction through the transition state is a hydrogen bond between substrate carboxylate and a threonine side chain hydroxyl (Thr 244, *E. coli* QFR numbering) (73). Because this catalytic threonine is on a different domain than the binding residues, interdomain motion changes the position of this hydrogen-bond donor, which twists the substrate during the reaction. Both a diode effect and the presence of a different flavin intermediate suggest that intermediates of succinate oxidation and fumarate reduction differ (71, 202). *C*, quinone/quinol interconversion. The second chemical reaction housed by complex II is the 2H^+^/2e^−^ interconversion of quinone and quinol in the membrane. In mitochondrial aerobic respiration, this involves the electrons harvested from succinate and uses ubiquinone as the quinone. Different types of respiration may use quinones with different potentials, which affects the driving force of the reaction. *D*, aerobic respiration usually relies upon oxidative phosphorylation to synthesize ATP because this is the most efficient way to convert energy to a biologically useful form. In animals, oxidative phosphorylation involves four membrane-spanning complexes of the electron transfer chain. The figure uses ovine complex I (*pink*, PDB 7ZD6, (208)), porcine complex II (*orange*, PDB 1ZOY (75)), bovine complex III (*green*, PDB 1NTZ (209)), and bovine complex IV (*blue*, PDB 2OCC (210)). These complexes guide the transfer of electrons down small, energetically favorable electron steps until they reach O_2_, which is reduced to H_2_O in complex IV. The strong oxidizing power of oxygen, ∼800 mV, drives the entire process. The overarching theme of all respiration is the coupling of these electron transfer steps with the formation of a transmembrane electrochemical gradient, shown as a proton gradient here. Complex V (*mustard yellow*, PDB 6ZPO (211)), also called the ATP synthase, converts the energy stored in the electrochemical gradient to a more biologically useful form. To do this, complex V couples the transfer of a proton back along its gradient with conformational changes that promote the formation of a bond between ADP and inorganic phosphate and that release the product, ATP. SDH, succinate dehydrogenase; QFR, quinol:fumarate reductase.

**Figure 2:**
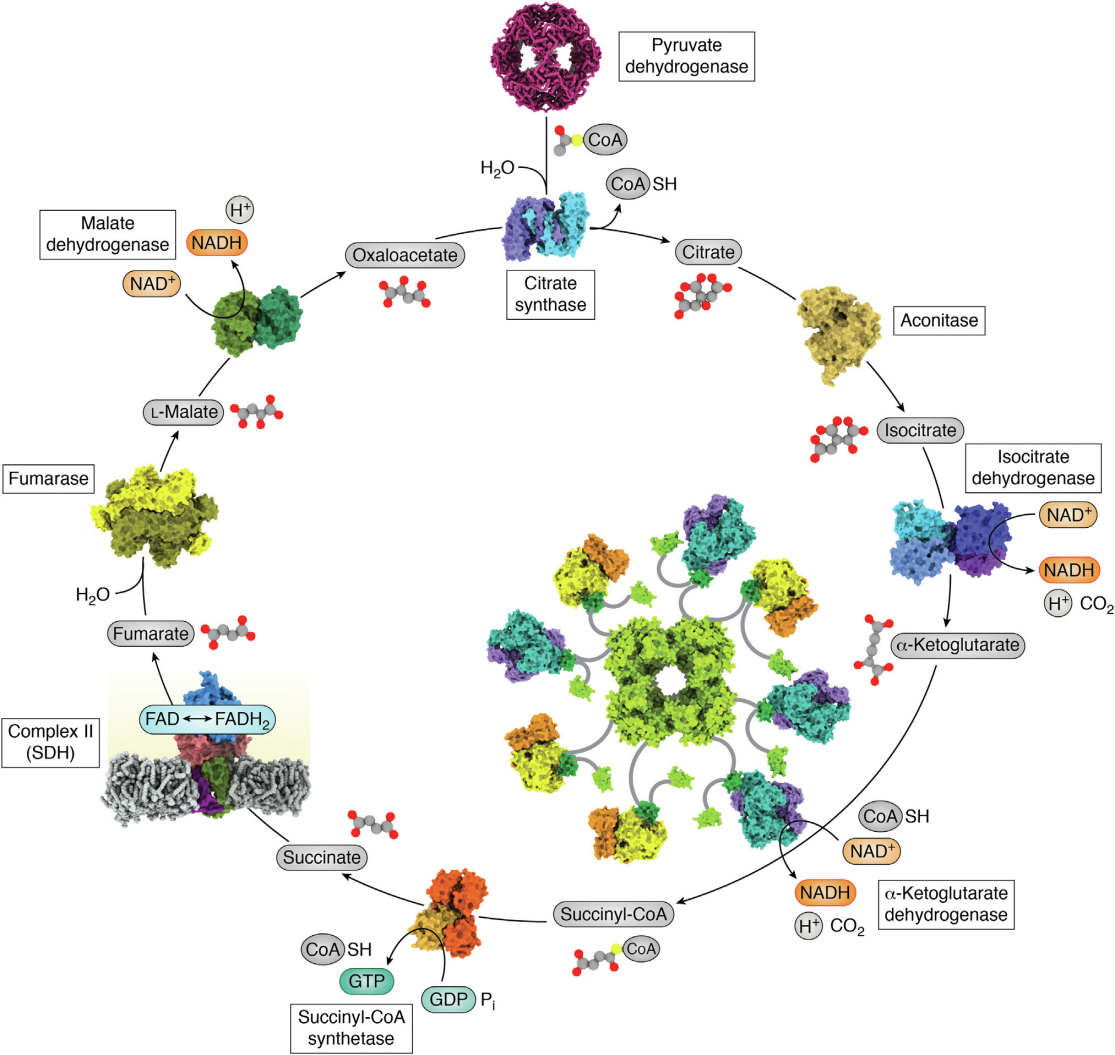
**Complex II in the Krebs cycle.** The Krebs cycle, also called the tricarboxylic acid cycle or the citric acid cycle, is an energy-harvesting process integral to aerobic respiration. The Krebs cycle uses a series of oxidation-reduction reactions on carboxylate-containing small molecules. The Krebs cycle contributes to oxidative phosphorylation in multiple ways. First, it produces reducing equivalents in the form of NADH, which is used by respiratory complex I. Second, the chemical intermediate succinate is used by complex II to provide another conduit for electrons to enter the respiratory chain. Coordinates used to develop this figure include human pyruvate dehydrogenase (PDB 6H55, (212)), porcine citrate synthase (PDB 1CTS, (213)), porcine aconitase (PDB 7ACN, (214)), human isocitrate dehydrogenase (PDB 7CE3, (215)), porcine succinyl CoA synthase (PDB 2FP4, (216)), porcine succinate dehydrogenase (PDB 1ZOY, (75)), human fumarase (PDB 5UPP, (217)), and porcine malate dehydrogenase (PDB 1MLD, (218)). The α-ketoglutarate dehydrogenase complex was modeled from the *E. coli* E1 component (PDB 2JGD, (219)), the *E. coli* E3-binding domain (PDB 1BBL, (220)), the *Thermus thermophilus* E3 component (PDB 2EQ7, (221)), and the *E. coli* lipoyl domain (PDB 1PMR, (222)). SDH, succinate dehydrogenase.

In addition to this respiratory role, complex II influences signaling pathways that control metabolism and cell fate (13). There may be multiple ways that complex II controls signaling. First, mature and assembled complex II can form direct complexes with effectors. For example, during bacterial chemotaxis, fumarate can induce a change in the direction of the bacterial flagellar motor, which in turn allows bacteria to reorient and change the direction of swimming. Complex II homologs that preferentially catalyze fumarate reduction directly bind to the flagellar motor and are essential for this chemotaxis with fumarate (14, 15, 16). In animals, complex II likely impacts signaling through the regulation of cellular levels of its substrate, succinate. The importance of succinate as a potent signaling molecule has become apparent over the past few years (13, 17). Complex II is *the* master controller of succinate levels in eukaryotic cells. As a result, changes in complex II activity and initiation of reverse activity (9, 10) each impact succinate signaling. Succinate signaling in eukaryotic cells proceeds *via* at least two distinct mechanisms. Succinate inhibits a range of enzymes *via* product inhibition when succinate is a coproduct of these enzymes (18, 19). Succinate can also stimulate succinate receptor 1 (SUCNR1, formerly GPR91), which is a promiscuous G protein–coupled receptor (GPCR) (20).

Of note, complex II subunits may affect signaling by participating in alternative assemblies (21, 22, 23, 24) (Fig. 3). SDHA is a stable part of a species that is termed “Complex II-low” (21) because it has a lower molecular weight than complex II. Complex II-low runs as a broad band on native gels, and it is not yet clear whether this is a single, defined species with unusual migration or whether it is a heterogeneous mixture of SDHA-containing species. To date, there are three stable SDHA-containing species that could be parts of complex II-low (Fig. 3*A*). Biologically, the accumulation of complex II-low correlates with altered signaling (21, 25).

**Figure 3:**
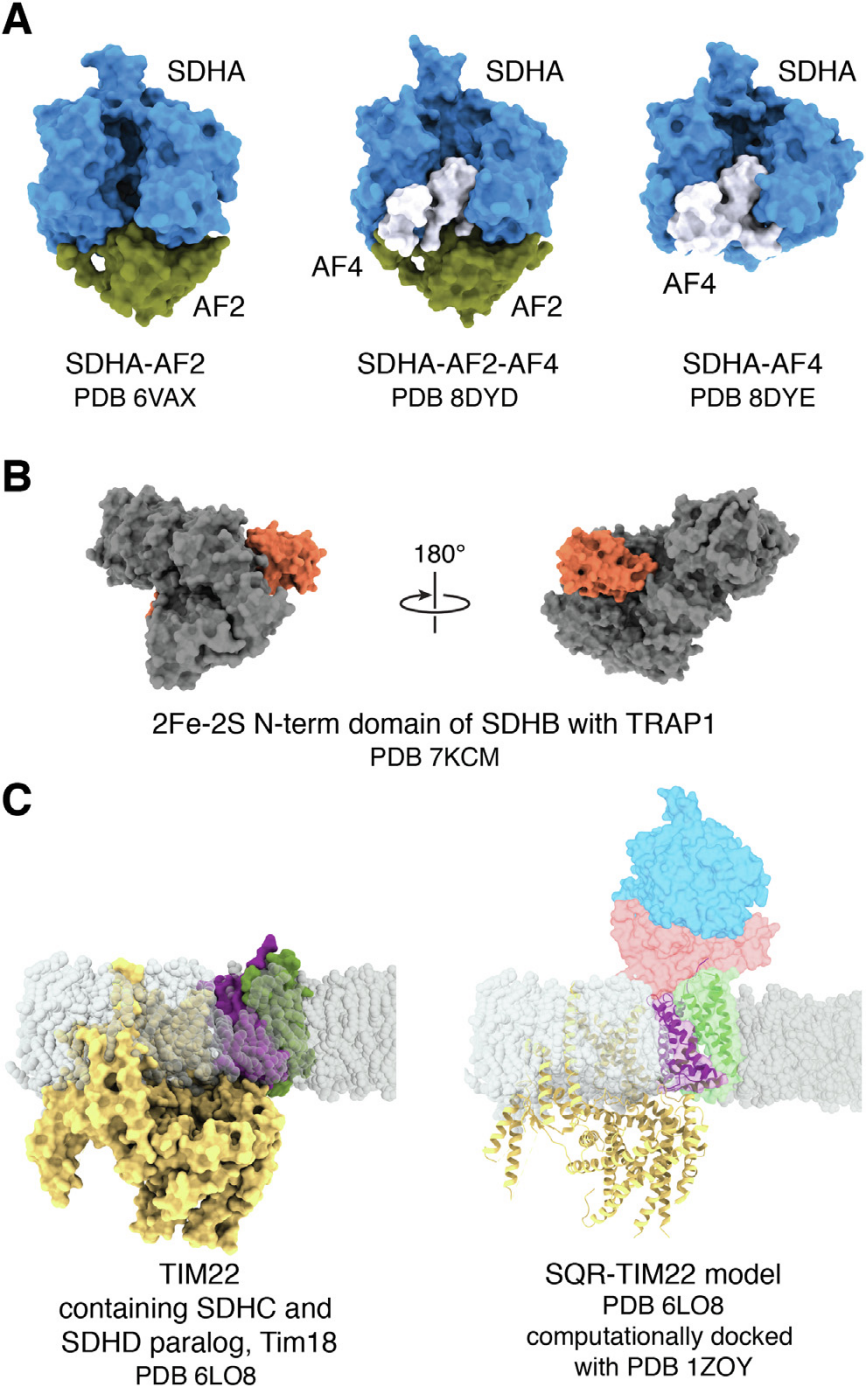
**Stable complexes that contain complex II subunits.** Complex II subunits have been shown to functionally integrate into alternative species. *A*, complex II-low is a heterogeneous mixture of species that contain SDHA, SDHAF2, and SDHAF4 that runs as a broad band on a native gel (21). The predominant species is likely the stable SDHA-AF2 complex (PDB 6VAX, (140, 142)). However, it cannot be excluded that complex II-low contains SDHA-AF2-AF4 (PDB 8DYD (140)) or SDHA-AF4 (PDB 8DYE (140)). *B*, the SDHB subunit forms complexes with TRAP1, which may have implications for regulating cancer metabolism (PDB 7KCM (133)). *C*, the SDHC subunit and an SDHD paralog called Tim18 (36% identical, 57% similar) form part of the mitochondrial inner membrane protein importer, TIM22 (22). The *left panel* shows the experimental structure, while the *right panel* shows a model with this docked to SDHA and SDHB subunits. Although sterics suggest that this complex could theoretically form, functional studies suggest that the Tim18 cannot support succinate-dependent respiration and SDHD cannot support TIM22 transport activity (22, 26). SDH, succinate dehydrogenase; SDHAF, succinate dehydrogenase assembly factor.

SDHA is not the only subunit found in alternative complexes. SDHC forms a membrane-spanning region of the yeast TIM22 protein importer that is required for function; here, SDHC interacts with an SDHD paralog called Tim18 (22, 24) (Fig. 3*B*). As a curiosity, yeast contains multiple paralogs of both SDHC and SDHD that are expressed under different respiratory conditions and have partial functional overlap (26). SDHD and the Tim18 paralog share 36% identity and 57% similarity and can both bind to SDHC, but they cannot functionally replace one another (26). Moreover, the SDHC-Tim18 heterodimer does not coordinate heme and therefore differs in cofactor status as compared to SDHC-SDHD. Finally, SDHB can be found in a complex with an HSP90 paralog called tumor necrosis factor receptor–associated protein 1 (TRAP1) (Fig. 3*C*); the stability of this complex and its functional significance is less clear (27, 28, 29, 30). One possibility is that some or all of these alternative complexes have distinct cellular roles. However, cellular succinate oxidation activity may be regulated by assembly and disassembly of the complex II heterotetramer (23, 31), and some of these complexes could also be storage forms for unassembled complex II subunits that are poised for rapid complex II assembly. As a result, these species may simply correlate with altered signaling.

Taken together, although complex II was among the first enzymes studied, its full biological role is still emerging. A semichronological consideration of findings in the field helps to highlight how the field pivoted in thinking about the roles for complex II in biology.

## Biochemistry, spectroscopy, and enzymology of mitochondrial enzymes

From the 1950s through the 1980s, most studies on complex II used mitochondrial enzyme purified from slaughterhouse material, with some studies using the yeast enzyme (32, 33, 34). These early studies not only investigated the details of complex II function but also used complex II as a model to understand the basic principles of enzymology and the role of cofactors in catalysis. Complex II has a number of features conducive to its use as a model system. These features include high-level expression, absence of cellular toxicity, and reasonable stability. Moreover, complex II has a hallmark visible absorbance spectrum imparted by its many cofactors: FAD, iron–sulfur clusters (2Fe-2S, 4Fe-4S, 3Fe-4S), and heme *b*. This spectrum translates to a reddish-brown color that can be followed by eye. Detailed early investigations assigned redox potentials to each of these species. Among the more important basic findings from this era was the discovery that the FAD cofactor could be covalently attached to protein (35, 36). In complex II, the covalent attachment to the protein raises the redox potential of the FAD cofactor by ∼100 mV (37). This increase in potential is required for the succinate oxidation reaction.

## A switch to bacterial model systems

Beginning in the 1980s, the complex II field shifted toward the use of bacterial model systems. One advantage of the bacterial enzymes is that, at that time, these more easily allowed the use of molecular biological techniques. However, because of differences in sequence conservation in different subunits of complex II enzymes, there are limitations on whether some findings can be extrapolated to the entire family. As an example, bacterial complex II enzymes all have detectable sequence similarity with mitochondrial complex II in the soluble SDHA and SDHB subunits (30%–50% identical, 50%–75% similar). However, many complex II homologs lack detectable similarity in the membrane-spanning subunits (38). These membrane-spanning subunits contain the quinone/quinol binding site and sometimes contain heme *b*. Even though work on bacterial complex II showed fundamental features of quinol chemistry (39, 40, 41) and how heme *b* could contribute to function (42), many investigations of bacterial complex II focused on the succinate oxidation reaction in the soluble domains.

The first mutagenesis studies on bacterial homologs were, in fact, instrumental in building a deep understanding of the enzymology of complex II (Fig. 1, *B* and *C*) (37, 43, 44, 45, 46, 47, 48, 49, 50). An additional early application of site-directed mutagenesis included the recapitulation of a patient mutation in the *Escherichia coli* complex II homolog (51, 52). A surprising discovery was that the substitution of active site residues resulted in the full elimination of succinate oxidation in the purified enzyme, with the clinical phenotype of optic atrophy, ataxia, and myopathy being milder than might be expected for the loss of a central metabolic enzyme (51, 52). Here, the patient was heterozygous, so had ∼50% of the normal level of complex II activity (51, 52). In fact, given that SDHD knockout in mice is embryonic lethal (53), heterozygous loss of complex II catalytic function *via* mutation often produces unexpectedly mild clinical symptoms with late onset, usually when the individual is in their 40’s (18, 54, 55, 56). Homozygous loss of complex II activity or activity below 50% of normal levels has been associated with earlier symptom onset and more severe symptoms (57).

## Membrane protein crystallography and structure-function approaches

**Figure 4:**
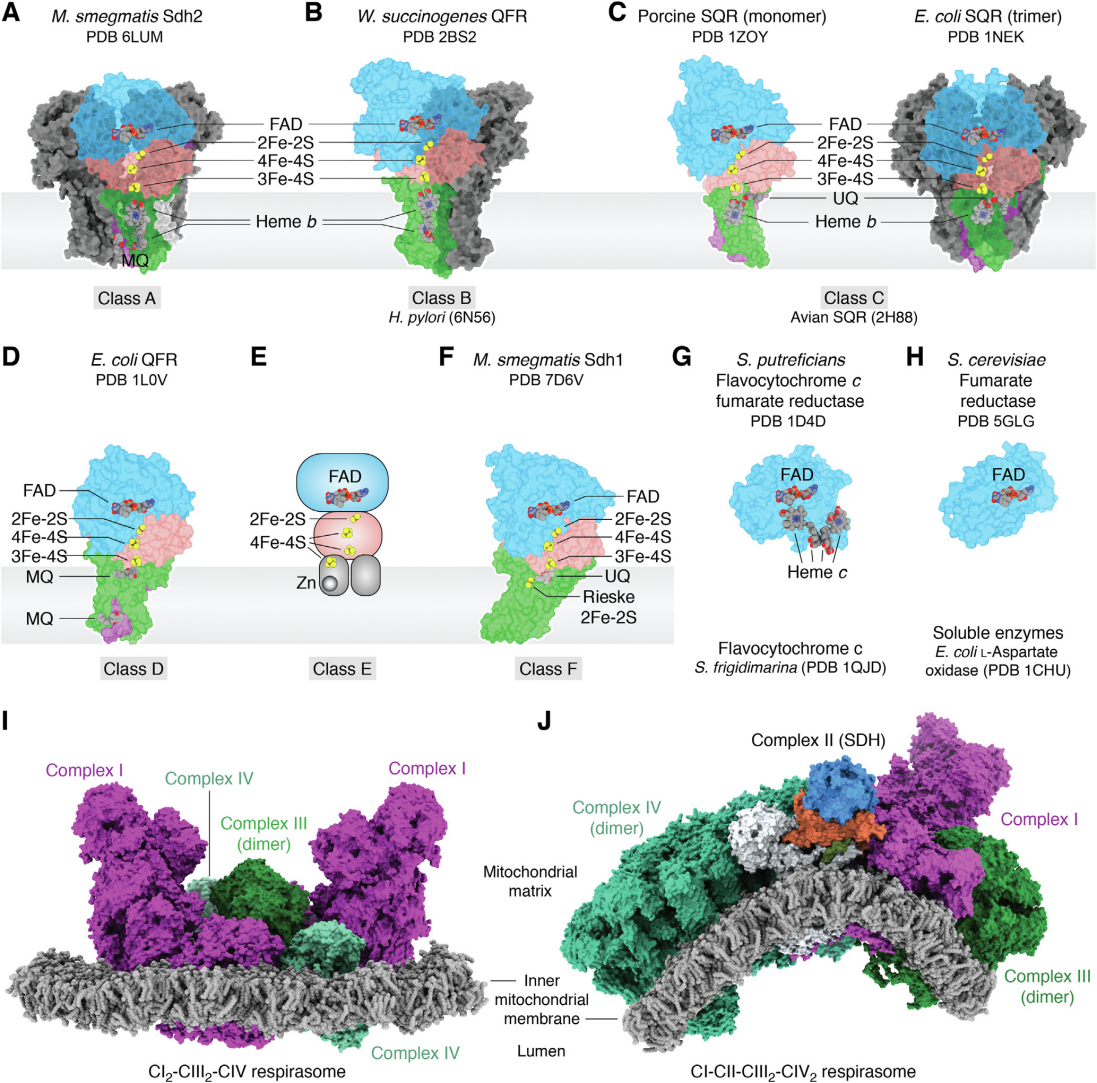
**Diversity in structure and megastructure across the greater complex II family.** Complex II enzymes are currently categorized into six recognized classes based on their membrane-spanning regions (83). Most members of the complex II superfamily contain a soluble domain with two subunits and between one and three membrane-spanning subunits, although Class B members are believed to peripheral-membrane enzymes and there are fully soluble homologs. While all complex II homologs share significant sequence identity in the soluble domains, the membrane-spanning domains may have evolved independently, possibly more than one time. In addition, some complex II family members contain supernumerary subunits. Depending on whether they were discovered for a physiological role in aerobic or anaerobic respiration, the corresponding complex II genes are termed *sdhABCD* (aerobic form) or *frdABCD* (anaerobic form). *A*, Class A complex II enzymes are predominantly from archaea. These can contain three membrane-spanning subunits, two of which have three membrane-spanning helices, with the additional single-pass membrane-spanning subunit termed SdhF. Class A homologs house two integral-membrane *b*-type hemes. Shown is the cryoEM structure of *Mycobacterium smegmatis* Sdh2, which is a trimer (PDB 6LUM (77)). *B*, Class B complex II contains a single membrane-spanning subunit with five membrane-spanning helices and two *b*-type hemes. Class B complex II enzymes are fumarate reductases and obligate dimers. Shown is the X-ray crystal *W. succinogenes* QFR (PDB 2BS2 (59)). *C*, Class C complex II enzymes contain two membrane-spanning helices and one *b*-type heme. Members of Class C complex II are of intense interest because they include mitochondrial homologs. Some Class C enzymes are (*left*) monomers (porcine mitochondrial complex II, PDB 1ZOY (75)) while others are (*right*) trimers (*E coli* SQR, PDB 1NEK (42)). *D*, Class D complex II enzymes are monomers that contain two membrane-spanning helices but no integral membrane heme. A putative cofactor is located at a similar position as heme in other homologs, but the identity of this has not been assigned (223). Shown is the *E. coli* QFR (PDB 1L0V (58)). *E*, Class E complex II enzymes contain membrane anchors with an amphipathic nature that suggests they could be peripherally associated with the membrane (224). Class E complex II enzymes coordinate a Zn^2+^ and 4Fe-4S cluster in the amphipathic helices. A structure representing this class has not yet been reported. *F*, Class F complex II enzymes contain a single membrane-spanning subunit with six helices. Members of Class F coordinate an integral-membrane Rieske Fe-S cluster. Shown is the structure of *Mycobacterium smegmatis* Sdh1 (PDB 7D6V (79)). *G* and *H*, soluble homologs of the SDHA subunit have distinct biological roles. *G*, some soluble SDHA homologs contain a fused tetra-heme domain and biologically act as fumarate reductases in anaerobic respiration (60, 61, 62). Shown is the *Shewanella oneidensis* (formerly *putrefaciens*) flavocytochrome *c* fumarate reductase (PDB 1D4D (62)). *H*, some soluble SDHA homologs are found without additional fused polypeptide and can catalyze redox reactions related to dicarboxylate oxidoreduction. The most extensively studied soluble SDHA homolog is perhaps L-Aspartate oxidase. Shown is the yeast Osm1 fumarate reductase (PDB 5GLG (94)). *I* and *J*, numerous structures of respiratory supercomplexes have been reported, with varying stoichiometries. These all contain complex III (CIII) at the core, which is an obligate dimer. Complex I (CI) and complex IV (CIV) can each interact with CIII independently and have one or two copies associated with each CIII_2_. *I*, reported respirasomes lacking complex II include: CICIII_2_, CIII_2_CIV, CICIII_2_CIV, and CI_2_CIII_2_CIV_2_. Shown is the human CI_2_CIII_2_CIV_2_ respirasome (PDB 5XTI, (110)) with CI in *magenta*, CIII in *green*, and CIV in *teal*. *J*, the only reported structure of a respirasome that contains complex II (CII) is from the ciliate *Tetrahymena thermophila* and has a stoichiometry of CICIICIII_2_CIV_2_ (80). Note that *Tetrahymena* respiratory complexes contain supernumerary subunits as compared to their counterparts in animals (111). This is particularly notable in CIV and in CII, with the latter containing 11 supernumerary subunits (SDHTT1 – SDTT11). For complex II, SDHA is in *blue*, SDHB is in *red*, SDHC and SDHD are in *green* and *purple*, and the 11 supernumerary subunits are in *white*. SDH, succinate dehydrogenase; QFR, quinol:fumarate reductase.

In the late 1990s, bacterial complex II was among the first membrane proteins with structures determined by X-ray crystallography (Fig. 4) (58, 59). Here, the interrogation of both soluble (60, 61, 62) and membrane-associated (58, 59) complex II homologs involved in bacterial anaerobic respiration led the field. Termed fumarate reductases or quinol:fumarate reductases, these complex II enzymes preferentially reduce fumarate to succinate (63, 64, 65, 66). This reaction is the reverse of that catalyzed during oxidative phosphorylation and the Krebs cycle. Once conditions for ready structure determination were identified, the application of a careful structure-function approach on mutant fumarate reductases allowed for a deep understanding of the mechanism of fumarate reduction (Fig. 1*B*) (67, 68, 69, 70, 71, 72, 73).

Structures of porcine and avian mitochondrial complex II from slaughterhouse sources were determined in the mid-2000s (74, 75). These additional structures were particularly important for revealing the details of the membrane-spanning regions of the mitochondrial enzymes. Structural studies with inhibitors helped to explain how quinone chemistry is supported in the mitochondria (74, 75, 76).

Recent advances in cryoEM have also been applied to members of the complex II family, allowing for structure determination of additional complex II enzymes without crystallization (77, 78, 79, 80). With this combination of crystallography and cryoEM, structures of complex II homologs have now been reported from a range of model organisms and pathogens. There are at least 14 unique complex II enzymes in the protein database. These enzymes reveal broad architectural diversity as they vary in oligomerization, the presence of supernumerary subunits, membrane protein composition, integral-membrane cofactors, and preferred quinone substrates (42, 58, 59, 74, 75, 77, 78, 80, 81, 82). These distinct complex II structures represent five of the six recognized evolutionary subclasses of complex II (Fig. 4, *A*–*F*) (83).

The broad structural variability across the membrane subunits allows for the development of class-selective inhibitors that target the quinone binding site. Notably, the ability of complex II to connect the Krebs cycle with respiration is critical for antimicrobial resistance, virulence, biofilm formation, and growth of many pathogens, including bacteria, fungi, and parasites (84, 85, 86, 87, 88, 89, 90). Complex II inhibitors have long been used as antifungal agents. Indeed, complex II inhibitors are currently among the fastest growing classes of agricultural antifungals (91). In addition, complex II inhibitors are being developed to target human pathogens. In this respect, the high-resolution structures of complex II, particularly those from *Ascaris suum* (81) and *Mycobacterium tuberculosis* (77, 79), have opened the possibilities for structure-based development of therapeutics for these difficult to treat pathogens (92, 93).

There are also structures available for soluble homologs of complex II; these enzymes do not catalyze the quinone/quinol half-reaction that is supported by the integral-membrane complex II enzymes. Soluble complex II homologs include flavocytochrome *c* fumarate reductases from *Shewanella* that contain a cytochrome domain (60, 61, 62) (Fig. 4*G*), soluble fumarate reductase from yeast that does not contain a cytochrome domain (94) (Fig. 4*H*), and L-Aspartate oxidase from *E. coli* (95, 96).

## Complex II as a part of megacomplexes

The increasing capacity to interrogate very large protein complexes now allow structures to be determined for respiratory megacomplexes (80, 97, 98, 99, 100, 101, 102, 103, 104, 105, 106, 107, 108, 109, 110, 111). Sometimes called respirasomes, these megacomplexes contain more than one respiratory complex. The close association between individual complexes may improve stability, allow for local coordination of respiration, or optimize the kinetics and efficiency of respiration by forming conduits to pass respiratory intermediates. Complex II is conspicuously absent from all (97, 98, 99, 100, 101, 102, 103, 104, 105, 106, 107, 108, 109, 110, 111) but one (80) of these reported respirasome structures. When it is present, complex II induces curvature in the respirasome due to its asymmetric shape (Fig. 4, *I* and *J*) (80). This gives rise to a hypothesis that complex II incorporation into the respirasome could stabilize the folds that hallmark the cristae. But this also suggests that most respirasomes do not contain complex II. The lower affinity of complex II for respirasomes may stem from how it interacts with the other proteins. Complex II has the smallest buried protein–protein interface of any of the species within the respirasome. Moreover, some of the complex II surface on the interior of the respirasome is prevented from making direct protein–protein interactions with the remaining complexes by a defined lipid pocket. This suggests that complex II is truly less strongly associated with respirasomes and implies that much of the complex II population is found outside these megacomplexes.

If the function of complex II is in the chemical reactions that contribute to respiration and signaling, why isn’t it more stably associated with the respirasome? And does it do something unique when it is not associated with the respirasome? One possibility is that complex II participates in other macromolecular supercomplexes. Inspiration for such a role is found in bacterial chemotaxis. Here, the anaerobic complex II homolog, fumarate reductase, binds directly to the flagellar switch complex (14, 15, 16). This interaction supports the assembly of the bacterial flagellum and is required for chemotaxis in response to fumarate (14, 15, 16). In mitochondria, initial reports suggest the existence of a Krebs metabolon (112, 113), which could contain complex II. A Krebs metabolon could increase the efficiency of the Krebs cycle by providing defined conduits for passing small molecule intermediates from one enzyme to the next (112, 113).

## Bioinformatics, disease-associated mutations, and altered cell fate

As the field matured in the early 2000s, an increasing number of noncanonical functions for complex II began to be reported. Consistent with this, a long-standing mystery in the field is that complex II deficiency is not associated with a defined set of patient symptoms. This also became clear in the early 2000s, when rapid advances in genome sequencing resulted in an explosion in the identification of disease-associated mutations within the complex II genes, which have been compiled into publicly accessible mutation databases (114).

A small number of mutations, particularly those associated with the SDHA subunit, are associated with different types of neurodegenerative disease. This disease phenotype mirrors what is observed with chemical inhibition of the SDHA subunit by the covalent inhibitor 3-nitropropionate (115). 3-nitropropionate induces specific loss of striatal neurons and has been used in animals to induce Huntington’s disease-like symptoms (115). Mutation of mitochondrial complexes commonly results in neurodegenerative phenotypes, with a theory that neurons have a disproportionately large requirement for oxidative phosphorylation. Indeed, chemical inhibition of complex I is used as a model for Parkinson’s disease as it results in the loss of motor neurons (116). Although it is not clear why the reduced function of electron transfer complexes disproportionately affects different populations of neurons, this could suggest that some mutations of complex II alter mitochondrial bioenergetics (Fig. 1*D*).

The overwhelming majority of disease-associated complex II mutations, however, are found in the SDHB, SDHC, and SDHD subunits (114). Individuals with complex II mutations in the SDHB, SDHC, or SDHD strongly correlate with many types of genetically linked endocrine neoplasia, in particular pheochromocytomas (38, 114, 117) and paragangliomas (118, 119, 120); more recent studies suggest that there must be additional mutations in one or more proteins outside of complex II in order for tumorigenesis to occur (121). Downregulation of SDHB, SDHC, or SDHD is also a common feature of clear renal cell carcinoma, with the loss of cellular SDH activity correlating with invasiveness (122, 123).

Complex II activity has long been known as a tumor suppressor (124) but the mechanism underlying this phenomenon remains less clear. Confounding the understanding of this process is that mutations in different subunits may result in cells with different propensities to promote metastasis (125, 126). Cancer cells disfavor oxidative phosphorylation and favor glycolysis, which is termed the Warburg effect, and complex II attenuates aerobic glycolysis. Although the role of complex II appears to be intimately linked with metabolism, one possibility is that the shift to neoplastic growth is triggered *via* a nonrespiratory function of complex II or by its substrate, succinate.

Much more recently, advances in genome editing have allowed for conditional knockout of complex II subunits in specific cell types of model animals. The range of observed phenotypes suggests that there may be functions of complex II that are not yet understood and can even sometimes show opposing phenotypes. For example, attempts to use a conditional knockout to create a mouse model for pheochromocytoma instead resulted in obesity (127). In two recently reported studies, mouse genetic models that affected complex II levels had opposing effects on lifespan (128, 129).

## Complex II assembly and the identification of new molecular players

Complex II activity may be at least partially regulated at the level of assembly. Understanding the processes of folding and assembly have been at the forefront of the field in recent years (130). It is now clear that complex II assembly requires both general and dedicated chaperones. The HSP60 chaperone has long been known to promote the folding of apo-SDHA within the mitochondrion (131). A second chaperone, TRAP1, has long been implicated in regulating mitochondrial metabolism (132) but has more recently been suggested as a chaperone for the SDHB subunit of complex II (27, 28), as supported by cryoEM snapshots of this process (133) (Fig. 3*C*). Notably, there are conflicting reports of how TRAP1 affects complex II activity. Some studies suggest that TRAP1 inhibits complex II and promotes neoplastic growth, and other studies show that TRAP1 is required for full complex II activity and promotes homeostasis (27, 28, 29, 30).

Dedicated assembly factors for the soluble SDHA and SDHB subunits of complex II were identified *via* genome sequencing of patients with complex II deficiency but no mutations in any of the complex II subunits (134). This led to the eventual discovery of four succinate dehydrogenase assembly factors (SDHAFs), termed SDHAF1–SDHAF4 (134, 135, 136, 137, 138, 139). Of these, SDHAF2 has homologs in all kingdoms of life, with bacterial homologs termed SdhE and yeast homologs termed Sdh5. In contrast, SDHAF4 is pan-eukaryotic with a limited number of homologs in bacterial α- and γ-proteobacteria that are not commonly used as model systems (140). As a result of the low number of bacterial models for SDHAF1, SDHAF3, and SDHAF4, studies of these complex II assembly factors combined work other models, particularly yeast, due to the ease of genetics. In yeast, these assembly factors are termed Sdh5–Sdh8 (134, 136, 139).

Roles for these assembly factors are still being explored. SDHAF2 and SDHAF4 act as assembly chaperones for the SDHA subunit. Of these, the better understood role is for the SdhE/Sdh5/SDHAF2 assembly factor, which works with dicarboxylate to enhance the attachment of the covalent FAD (134, 141, 142, 143, 144, 145). While the bacterial FrdA-SdhE and SdhA-SdhE complexes have little buried surface area and can readily disassociate (146, 147), the human SDHA–SDHAF2 complex (Fig. 3*A*) is highly stable, long-lived, and appears to accumulate in cancer cell lines (21). The role of SDHAF4 (yeast Sdh8) is more cryptic, but it releases SDHAF2 from the SDHA–SDHAF2 complex (140), which is a requirement for the assembly of functional complex II (136, 137, 148). SDHAF1 and SDHAF3 are proposed to act as chaperones for Fe-S assembly within the SDHB subunit (138, 139). How these assembly factors act and whether there is any interplay with SDHAF4 or TRAP1 remains to be reported.

Finally, there are currently no reports of assembly mechanisms or dedicated chaperones for the integral-membrane SDHC and SDHD subunits. Heme *b* insertion into the membrane-spanning domain would be required for the full function of complex II. As mechanisms of assembly is a rapidly advancing area of research, more may be known in the future.

## Succinate signaling, regulation of transcription, and cell fate

As recently as 2010, advances in cell biology were leveraged to show that the activity of complex II controls cell fate in a way that does not strictly result from a primary metabolic function. For example, the inducible loss of complex II activity is linked to a number of ROS- or succinate-dependent (149) biological activities, including DNA methylation, inflammation, cell fate in adipocytes, and cancer metabolism (18, 54, 55, 56). It is now appreciated that some types of cellular signaling are linked to complex II activity.

To understand the molecular mechanisms underlying how complex II signals, the synthesis of key relevant results that were reported before a signaling role was considered in the field is important. Among the earlier curiosities was the discovery that hypoxia or cellular acidification in response to anticancer drugs could cause the specific disassembly of complex II and the accumulation of a ∼100 kDa soluble species containing the SDHA subunit (23). These soluble species were interpreted as an SDHA–SDHB complex (23), but more recent work in macrophages showed that species observed upon complex II disassembly do not contain SDHB (31). Although the species was not assigned in either work, one possibility is that complex II disassembly species might be a complex between SDHA and an assembly factor. The SDHA-AF2 species (142) has recently been suggested as the dominant molecular component of this species, with this complex being highly stable and migrating at an appropriate molecular weight (150). Despite the significant accumulation of this ∼100 kDa species in cells under these conditions, no independent biological activity of the purified SDHA-AF2 complex has been detected (150). One possibility is that this ∼100 kDa species may be a way to store SDHA subunits in an inactive state following complex II disassembly while poising the cell for rapid reassembly when metabolic conditions change. No matter the nature of the accumulated species, the loss of assembled complex II and the appearance of the ∼100 kD SDHA-containing species correlate with reactive oxygen species generation, increased apoptosis, and the accumulation of the complex II substrate, succinate, in cells (23, 31). One interpretation of this is that dynamic assembly and disassembly of complex II regulates succinate signaling.

**Figure 5:**
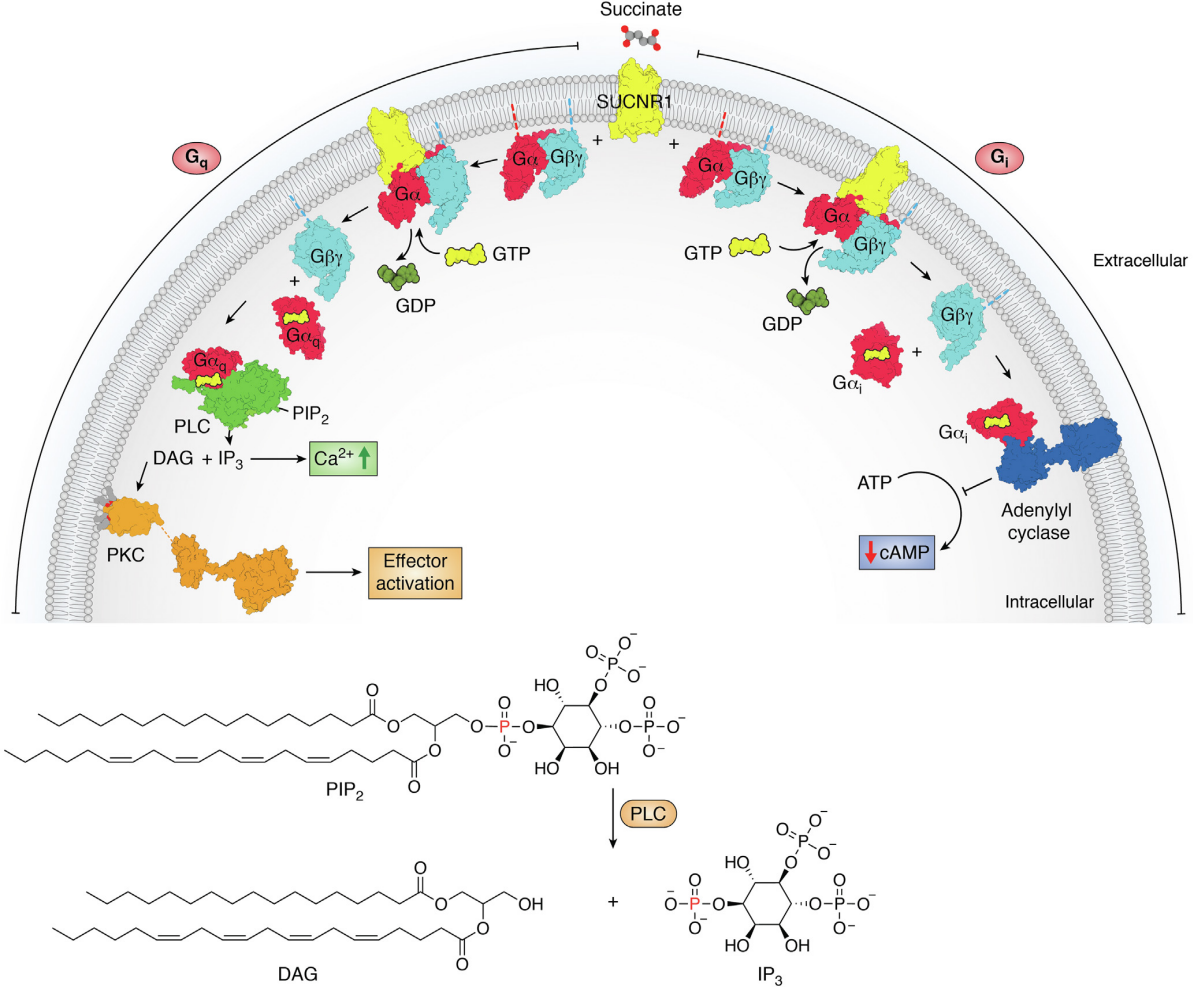
**Succinate-stimulated SUCNR1 signaling.** SUCNR1 is a GPCR, or 7-TM receptor, that responds to stimulation by succinate (20). Like other GPCRs, SUCNR1 and the proteins in its downstream signaling pathways transmit information through conformational changes that can create or remove binding sites and/or change the catalytic activity of effectors. Although the pharmacology and signaling pathways are still in the process of being mapped, SUCNR1 (PDB 6RNK (225)) has been shown to couple to either G_q_ or G_i_. In the figure, the SUCNR1-G protein complex is modeled from 6RNK (225) and either 1GOT (226) or 3SN6 (227), using 3SN6 as a guide to place the Gα and Gβγ subunits. Both G_q_ (modeled from 2BCJ (228) and 1GOT (226)) and G_i_ (modeled from 1GIA (229) and 1GOT (226)) are heterotrimeric G proteins containing Gαβγ subunits. SUCNR1 initiates G protein-dependent signaling by catalyzing GDP release from the Gα subunit. The binding of GTP, which is at a higher concentration in the cell, disassociates the GPCR–Gαβγ complex, releasing GTP-bound Gα (2BCJ (228) or 1GIA (229)) and Gβγ (from 1GOT (226) or 1TBG (230)). Classical Gα_q_ (from 2BCJ (228)) signaling involves the activation of phospholipase C (PLC, 7SQ2 (231)), which catalyzes the cleavage of the membrane-associated PIP_2_ into two potent signaling molecules: IP_3_, which is soluble, and diacylglycerol (DAG), which remains membrane-attached. IP_3_ mobilizes intracellular Ca^2+^ while DAG activates protein kinase C (PKC, modeled from PDB 3PFQ (232) and PDB 7L92 (233)) *via* a C1 domain. The kinase activity of PKC leads to the activation of numerous signaling effectors. Among the most potent and best characterized effectors are the Ras-Raf-MEK-ERK signaling cascade, but other mitogen activated protein kinases (MAP kinases), and Src family kinases are downstream of PKC. Signaling *via* Gα_i_ (PDB 1GIA (229)) inhibits adenylyl cyclase (AC, modeled from PDB 1CJK (234)), which normally converts ATP to cyclic AMP (cAMP). The inhibition of adenyl cyclase, therefore, decreases cAMP, which has a range of effects on cAMP-dependent processes. GPCR, G protein-coupled receptor; SUCNR1, succinate receptor 1.

Indeed, within the last 15 years, metabolites including succinate and other Krebs cycle intermediates began to be recognized as signaling molecules (13, 17, 149, 151, 152). Succinate signaling occurs *via* more than one distinct molecular pathway. One route of signaling by succinate and other Krebs cycles intermediates is receptor-mediated. Here, accumulated cellular succinate stimulates the GPCR, SUCNR1, which is differentially expressed in both immune and adipose cells (153), including in the renin-producing cells of the nephron. This effect is currently believed to be local rather than systemic. SUCNR1 can couple to both G_i_ and G_o_ (154, 155, 156) and to activate a range of downstream effectors *via* canonical G protein–dependent pathways (Fig. 5); SUCNR1 does not strongly couple arrestins and has not been demonstrated to initiate arrestin-dependent signaling. Many of the effectors downstream of G_i_/G_q_, like mitogen-activated protein kinases, Src-family kinases, and phosphoinositol kinases promote cellular survival, growth, proliferation, and metastasis (157, 158, 159, 160, 161). Potentially due to the activation of these pathways, SUCNR1 stimulation by succinate has been linked to several types of cancers. SUCNR1 may be involved in a number of succinate-associated health effects including ischemia-reperfusion injury, hypertension, inflammation, rheumatoid arthritis, nonalcoholic fatty liver disease, angiogenesis, and exercise-induced muscle remodeling (162, 163, 164, 165, 166, 167, 168, 169). These SUCNR1-dependent processes could explain how complex II, which controls cellular levels of the agonist, succinate, is the only respiratory enzyme that regulates metabolism or acts as a tumor suppressor (170, 171). Notably, GPCRs tend to be excellent pharmacological targets. Given the broad number of biological effects and diseases that depend upon succinate signaling through SUCNR1, this receptor has been aggressively pursued as a potential therapeutic target (172, 173, 174, 175) since its deorphanization (20).

Cellular succinate, and other Krebs cycle intermediates with a similar chemical structure, can inhibit enzymes where succinate is a product. This has long been known to regulate the Krebs cycle *via* feedback inhibition. Succinate is also a coproduct of a very large number of α-ketoglutarate-dependent enzymes ((176); also called 2-oxoglutarate-dependent enzymes) and can inhibit these enzymes. Among enzymes affected by Krebs cycle intermediates are members of the nonheme iron α-ketoglutarate-dependent superfamily. Although other Krebs cycle intermediates, such as fumarate, have a higher affinity for the nonheme-iron α-ketoglutarate-dependent enzymes than does succinate (176, 177), succinate is currently believed to be a physiological regulator due to its cellular concentrations.

**Figure 6:**
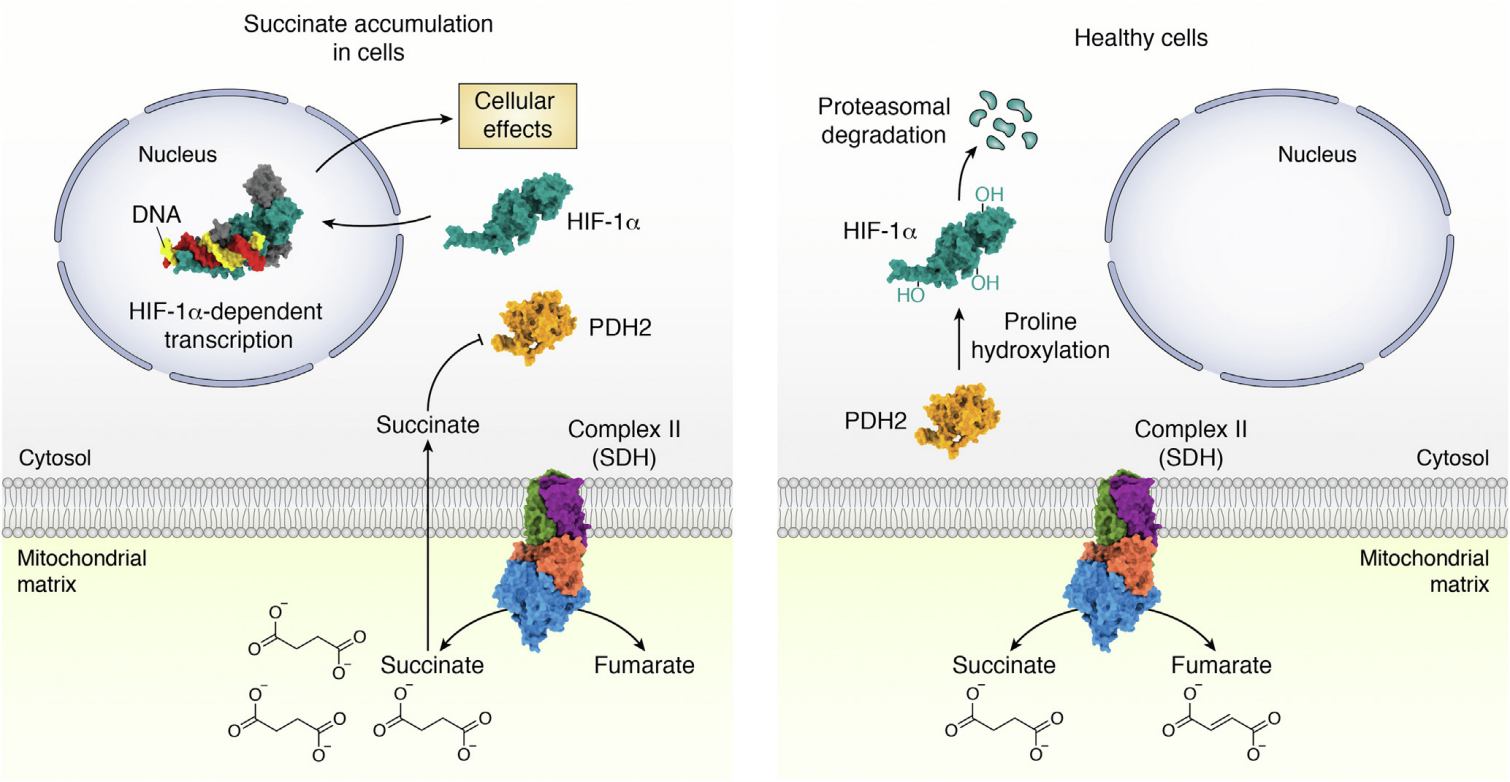
**Control of HIF1α activity through substrate-level inhibition of its degradation.** Healthy cells under normoxia use oxidative phosphorylation to synthesize the majority of ATP. During succinate accumulation, cells can redirect metabolism to generate lactate *via* glycolysis, which is less efficient in producing ATP. One metabolic switch involves changes in complex II (1ZOY (75)) activity, resulting in the accumulation of cellular succinate. Some of the biological situations where succinate might accumulate are hypoxia, during complex II inhibition, in patients with reduced functional complex II, during reverse electron transfer, or during cancer. One theory is that succinate accumulation controls the quantity of the transcription factor HIF1α (modified from 4ZPR (235)). In healthy cells under aerobic conditions, PHD2 (2CGN (177)) covalently attaches hydroxides to proline residues in HIF1α. The end effect is targeting of the HIF1α transcription factor for degradation by the proteasome. Cellular dicarboxylates, including succinate, inhibit prolyl hydroxylase-2. When HIF1α accumulates, it binds to DNA (4ZPR (235)) and induces the transcription of a range of proteins used in the hypoxic response. This shunts respiration away from oxidative phosphorylation and toward glycolysis and the biosynthesis of nucleotides and lipids. When this occurs under aerobic conditions in cancer cells, it is also termed the Warburg effect. HIF1α, hypoxia-inducible factor 1α; SDH, succinate dehydrogenase; PHD2, prolyl hydroxylase-2.

Examples of nonheme iron α-ketoglutarate-dependent dependent enzymes that have been demonstrated to be impacted by complex II activity are prolyl hydroxylases; these enzymes function to hydroxylate proteins to target them for degradation (Fig. 6). One substrate of prolyl hydroxylase-2 (PHD2) is hypoxia-inducible factor 1α (HIF1α) (178, 179, 180, 181), which is a master regulator of metabolism and is implicated in cancer metabolism. Thus, the accumulation of cellular succinate and increase of the cellular succinate:α-ketoglutarate ratio in cells following the reduction, loss, or reversal of complex II activity can inhibit PHD2, stabilize HIF1α, shift metabolism toward aerobic glycolysis (18), and induce cytokines and inflammation (182). This has broad implications for human health. As just one example, recent studies provide indirect evidence that succinate-dependent cytokine response may be a central feature of the severe- and long-COVID that hallmarks a subset of SARS-CoV2 infections. In fact, there is some overlap between the symptoms of complex II deficiency and some of the unusual symptoms of COVID (183, 184, 185). Cell-permeable diethyl succinate boosts viral load (186), while metabolomics identified that succinate accumulation profoundly increases with disease severity (187).

There are also other α-ketoglutarate-dependent enzymes in the cell affected by complex II activity. Notable among these are histone and DNA demethylases. Succinate-induced inhibition of these enzymes attenuates demethylation activity. This correlates with DNA hypermethylation (54) and epigenetic gene silencing, as confirmed by methylome and transcriptome analyses (188). The implications are far reaching because demethylases impact the transcription of genes involved in disparate processes and have epigenetic control over cellular pluripotency, cancer metabolism, cellular transformation, and macrophage activation (189, 190, 191, 192, 193, 194, 195, 196, 197). It should be noted that α-ketoglutarate itself, which could out-compete succinate, is reported to have opposing effects on cellular pluripotency *versus* differentiation (198, 199). The similarity of the processes triggered *via* inhibition of histone and DNA methylases and those triggered by SUCNR1 and HIF1α inhibition suggests synergy and functional overlap in the different succinate-dependent signaling cascades.

Finally, covalent succinylation (200, 201) is a posttranslational modification that can modulate the activity of biological macromolecules. Protein succinylation involves destination lysine residues that are also targets of acetylation (200, 201) and requires a succinyl-CoA donor rather than succinate itself (201). While this is therefore distinct from true succinate signaling, changes in complex II activity affect cellular succinate-CoA levels and may also affect succinylation.

These signaling functions have renewed interest in whether the fundamental biological role of complex II is fully understood. One recent area of intense focus has been complex II reversibility in the mitochondria (9, 10). Although all enzymes theoretically can perform bidirectional catalysis, *in vitro* work shows that both bacterial and mitochondrial complex II perform catalysis *via* different intermediates in the forward *versus* reverse directions (71, 202). It was therefore surprising that two groups independently reported that mitochondrial complex II could biologically reverse its direction and reduce fumarate to succinate (9, 10). This allows fumarate to replace O_2_ as the terminal electron acceptor in the electron transport chain and is an extremely inefficient way to support bioenergetics. Reverse complex II activity may also be part of anaerobic mitochondrial production of hydrogen (203).

Some aspects of reverse electron transfer seem to satisfyingly connect the enzymatic and signaling functions of complex II. However, other aspects bring new questions to the forefront. For example, the ability to use reverse electron transfer in mammals appears to be tissue-dependent, with kidney, liver, and brain more strongly supporting reverse electron transfer (10). Of these, the liver is the most likely to experience anaerobic conditions that might require a shift in metabolism.

## Integration over time: an evolving view

Synthesis of the findings on complex II reveals a shifting view over time. At present, it is now appreciated that there may be major roles for complex II in both respiration and in metabolic signaling. A particularly compelling theory is that complex II, or its individual subunits, contributes to multiple biological processes. This could potentially occur *via* direct protein–protein interactions in noncanonical complexes. Complex II may also move into and out of the megacomplexes during a response to the metabolic state.

Why would complex II have such diverse functions and such plasticity in its structural assemblies? Complex II is an evolutionarily ancient enzyme with ancestors that likely supported respiration before oxygen became abundant on the planet (83). Phylogeny hints that major changes in complex II rapidly occurred around the time of the great oxidation event (2, 204, 205). Perhaps the most important adaptation was the development of a covalent attachment between the FAD cofactor and the protein (35). The SDHAF2 assembly factor that enhances FAD attachment also likely appeared around the time of the great oxidation event (206). SDHAF2 moonlights as a component of the SDHA-AF2 species (142) of complex II-low, which is associated with altered metabolic signaling in cancer cell lines (21). Curiously, while SDHAF2 is found in all kingdoms and in most organisms that have a complex II homolog, the other characterized assembly factors are less broadly distributed.

While speculative, one could consider whether ancient cellular organisms might have benefitted from proteins supporting more than one biological function. It could further be considered whether metabolic signaling was among the earliest signaling systems. The present evidence that complex II connects metabolism and signaling could have assisted an ancient organism in its primordial need to swim toward food. The stable complex II scaffold and its subunits still serve these functions, amongst others. This change in conceptualization brings us one step closer to explaining the range of observations associated with complex II activity. It also directs the field to continually reassess whether we truly know the full role of complex II, or other proteins, in biology.

## Conflict of interest

The authors declare no conflicts of interest with contents of this article.
